# Freedom of choice adds value to public goods

**DOI:** 10.1073/pnas.1921806117

**Published:** 2020-07-13

**Authors:** Lei Shi, Ivan Romić, Yongjuan Ma, Zhen Wang, Boris Podobnik, H. Eugene Stanley, Petter Holme, Marko Jusup

**Affiliations:** ^a^Statistics and Mathematics College, Yunnan University of Finance and Economics, Kunming 650221, China;; ^b^Interdisciplinary Research Institute of Data Science, Shanghai Lixin University of Accounting and Finance, Shanghai 201209, China;; ^c^Center for OPTical IMagery Analysis and Learning, Northwestern Polytechnical University, Xi’an 710072, China;; ^d^Graduate School of Economics, Osaka City University, Osaka 558-8585, Japan;; ^e^School of Mechanical Engineering, Northwestern Polytechnical University, Xi’an 710072, China;; ^f^Center for Polymer Studies, Boston University, Boston, MA 02215;; ^g^Faculty of Civil Engineering, University of Rijeka, 51000 Rijeka, Croatia;; ^h^Zagreb School of Economics and Management, 10000 Zagreb, Croatia;; ^i^Luxembourg School of Business, 2453 Luxembourg, Luxembourg;; ^j^Faculty of Information Studies in Novo Mesto, SI-8000 Novo Mesto, Slovenia;; ^k^Tokyo Tech World Research Hub Initiative, Institute of Innovative Research, Tokyo Institute of Technology, Tokyo 152-8550, Japan

**Keywords:** social dilemma, cooperation, behavioral phenotypes, preferences, social networks

## Abstract

Public goods, ranging from judiciary to sanitation to parkland, permeate daily life. They have been a subject of intense interdisciplinary study, with a traditional focus being on participation levels in isolated public goods games (PGGs) as opposed to a more recent focus on participation in PGGs embedded into complex social networks. We merged the two perspectives by arranging voluntary participants into one of three network configurations, upon which volunteers played a number of iterated PGGs within their network neighborhood. The purpose was to test whether the topology of social networks or a freedom to express preferences for some local public goods over others affect participation. The results show that changes in social networks are of little consequence, yet volunteers significantly increase participation when they freely express preferences. Surprisingly, the increase in participation happens from the very beginning of the game experiment, before any information about how others play can be gathered. Such information does get used later in the game as volunteers seek to correlate contributions with higher returns, thus adding significant value to public goods overall. These results are ascribable to a small number of behavioral phenotypes, and suggest that societies may be better off with bottom-up schemes for public goods provision.

## Significance

Public goods, from tangible properties to intangible services, benefit all. They are produced or maintained through widespread participation in public goods provision. Low participation rates are therefore a looming threat that has motivated countless searches for ways to elicit participation. Recent theory suggests that social networks, as woven by personal relationships, are instrumental. We organized a social dilemma game experiment to investigate whether player participation in public goods provision depends on the global characteristics of social networks or the ability to freely choose among local public goods within a player’s network neighborhood. Our results demonstrate the importance of the latter factor, thus favoring bottom-up public goods provision that gives individuals a say in decision-making.

Judiciary, sanitation, and parkland, but also more abstract categories such as knowledge, herd immunity, and climate, are thought to be public goods (1). They are created or maintained through profitless society-wide engagement to the benefit of all. Pervasiveness of public goods makes them a subject of intense interdisciplinary study that often resorts to a multiplayer generalization of the famous prisoner’s dilemma (2) called the public goods game (PGG). The basic PGG variant comprises a number of players with endowments to be contributed toward a public pool in a series of game rounds. The total contribution in each round is multiplied by a compounding factor and then distributed equally to all players, yet the amount that each player contributes is unknown to others. Therein lies the dilemma, because players have an incentive to free ride by contributing little themselves while benefiting from the public pool. Players who free ride are therefore called defectors, whereas contributors are known as cooperators.

Theory and practice of PGGs are somewhat at odds (3). A rational player should try to maximize their own benefit by defecting, while hoping to benefit from the public pool to which others contribute. If all players are rational, nobody contributes, creating a stalemate called the Nash equilibrium in which no player can gain by a unilateral change of strategy. Despite such a dreary prediction, experiments show that contributions in one-shot PGGs hover around 50%, and the Nash equilibrium is approached, but not reached, only in iterated PGGs (4, 5). An ongoing debate attributes this behavior, rather pessimistically, to confusion about game rules or, more optimistically, to player prosocial tendencies (6–10). Although the debate is far from settled, it did not dissuade attempts to promote cooperativeness in PGGs via various additions. These include punishment, reward, transparency, reputation, heterogeneous endowments, and many others (11–16).

One common question regarding social dilemmas has been what the effect of the underlying interaction structure is. Typically, this has been answered by confining players to social networks that encode who interacts with whom. Examples range from highly stylized two-dimensional lattices (17) to more realistic small-world or scale-free network models (18, 19). Networked populations have been shown to support cooperation even when well-mixed ones cannot (20), particularly if a network is highly heterogeneous (21). In PGGs too, players with heterogeneous social ties provision to public goods more than their counterparts with homogeneous ties (22). Interestingly, large-scale experiments on the spatial prisoner’s dilemma failed to support these theoretical results, as cooperativeness in heterogeneous and homogeneous networks remained similar (23).

**Fig. 1.**
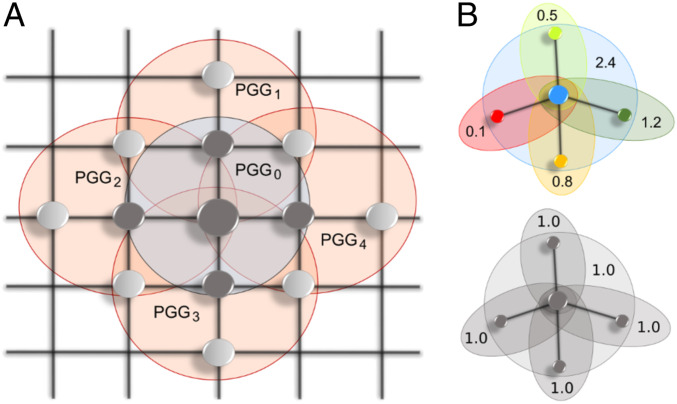
Schematics of our social dilemma experiment interweaving public goods games and social networks. (*A*) Taking lattice as an example, and focusing on the player in the center, we see that this focal player has four neighbors with whom they engage in a public goods game labeled ${PGG}_{0}$. However, to treat all players on an equal footing, each of the four neighbors is also a center of one game, labeled ${PGG}_{1}$ to ${PGG}_{4}$. The focal player takes part in all these games. The three social networks used in this study are visualized in *SI Appendix*, Fig. S1. (*B*) Under the free-contribution scenario (*Upper*), also referred to as experimental treatment, a player who decides to participate in public goods provision can freely decide how to distribute contributions among each of the public goods within their reach. A player’s reach is determined by the underlying social network. Under the fixed-contribution scenario (*Lower*), also referred to as experimental control, players can only decide whether or not to participate in public goods provision. Once they decide to participate, contributions are distributed equally among all reachable public goods.

To summarize the current situation, a traditional focus on participation in public goods provision in isolated PGGs (one-shot or repeated) has gradually shifted toward the role of social networks in enticing participation. We brought the two focal points together by arranging volunteers in a network of social ties upon which each volunteer took part in several PGGs along their specific ties (Fig. 1). We examined three network configurations: lattice, random regular network of degree 4, and random network in which half the nodes had degree 3 and the other half degree 5 (*SI Appendix*, Fig. S1). Each player acted both as a network node and a center of one PGG involving themself and their first neighbors (Fig. 1*A*). The number of PGGs that a player thus played was equal to one (a PGG centered around the player) plus their degree (PGGs centered around each of the first neighbors). With the described setup, we could detect how the topology of social networks affects participation in public goods provision. We could also separate participation (whether to contribute or not) from actual contributions (how much and where to contribute), thus examining decision-making when contributions are freely directed to preferred local public goods (Fig. 1*B*).

To perform the experiment, we recruited 596 student volunteers (63% women, mean age 19 y) at the Yunnan University of Finance and Economics in Kunming city, China (*SI Appendix*, Table S1). We distributed these volunteers across six experimental setups, which covered the three mentioned network configurations and two types of contributions, fixed or free. Varying the network configuration enabled us to test how topology influences actions. We accounted for 1) social compactness by comparing a lattice to a random regular network of the same degree because the former has a larger diameter than the latter, and 2) social connectedness by additionally organizing gameplay on a random network with the same average degree as the other two networks. Furthermore, separating the decision on whether to participate in public goods provision from the decision on how to allocate contributions enabled us to test how freely expressing preferences for certain local public goods over others affects behavior. Each setup was replicated twice for a total of 12 sessions of the game experiment organized between May and December 2018. During a session, volunteers engaged in gameplay via a custom computer interface (*SI Appendix*, Figs. S2 and S3). We incentivized better gameplay with a monetary payout at the end of each session. Further methodological information is available in *Materials and Methods* and *SI Appendix*, *Supplementary Methods*.

Our experiment demonstrates that freeing contributions to reflect individual preferences promotes participation in public goods provision. Compared to when contributions are fixed, players jump-start participation from the very beginning to form a cooperative environment that is independent of topology. Participation is subsequently held at significantly higher levels for prolonged periods of time as players seek to correlate their contributions with higher returns. Ultimately, players end up with greater wealth, thus proving that the freedom of choice adds value to public goods.

## Results

**Fig. 2.**
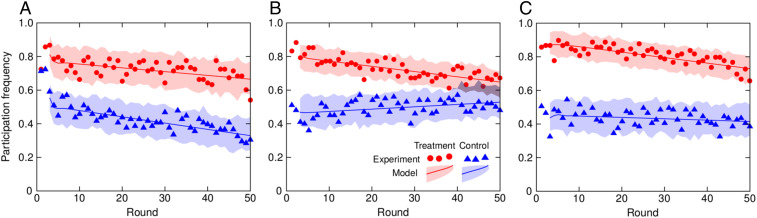
Participation in public goods provision leaps upward when contributions are freely chosen. Surprisingly, this leap happens in the very first round of the game before any information about the behavior of neighbors can be collected. Shown is the time evolution of participation in game experiments, with each dataset being the average of two replicates. Also shown is the average and the 95% confidence bands of a time series model fitted to the data (*SI Appendix*, Table S2). In control, players could only choose whether to participate or not, whereas, in treatment, they could also prioritize public goods of their preference. (*A*) In 7 × 7 lattice, the participation time series are downtrending in both treatment and control. (*B*) In regular random network, the participation time series is downtrending in treatment, but uptrending in control. (*C*) In degree 3 vs. degree 5 network, the participation time series is downtrending in treatment, but trendless in control. In all cases, the participation time series are stationary and nonautocorrelative. A negative trend in participation is common in PGGs, but not a general rule in our experiments, and considerable variation exists between individual replicates (*SI Appendix*, Fig. S4). A complementary statistical analysis in terms of a generalized linear mixed model (GLMM) is in Table 1.

The freedom to choose contributions in accordance with one’s own preferences increases participation in public goods provision regardless of social network configuration (Fig. 2 and Table 1). This result, while intuitive, arises in a surprising manner. The increase in participation happens instantly, from the first round of the game experiment, and without any transient dynamics (Fig. 2). In control, participation thus hovers around 50%, which is consistent with the previous PGG experiments (4, 5), but, in treatment, when players are free to choose where to contribute, participation jumps to above 80% irrespective of network configuration. That there is no transient dynamics to the state of increased participation is surprising because the freedom of choice provides an opportunity to gradually optimize behavior based on past experience. Although players could first learn which public goods are more productive, and only then direct contributions accordingly, this is not what transpires in the experiment. The initial increase in participation is nonetheless maintained for prolonged periods of time due to a more cooperative environment brought about by free choice.

**Table 1.** GLMM quantifies how participation in public goods provision differs between experimental setups

Row	Explanatory variable	Type	Coefficient	SE
0	Constant term	—	1.5464***	(0.1766)
1	Round	Integer	−0.6264***	(0.1126)
2	Random regular	Indicator	−0.1109	(0.2154)
3	Degree 3 vs. degree 5	Indicator	0.1695	(0.2165)
4	Control	Indicator	−1.5893***	(0.1765)
5	Round × random regular	Interaction	−0.2308	(0.1565)
6	Round × degree 3 vs. degree 5	Interaction	−0.2925	(0.1745)
7	Round × control	Interaction	−0.3229*	(0.1531)
8	Round × random regular × control	Interaction	1.4297***	(0.2087)
9	Round × degree 3 vs. degree 5 × control	Interaction	1.0203***	(0.2306)

Taking treatment in lattice as a reference, we see that participation in public goods provision exhibits a significant negative trend (row 1). Other coefficients are adjustments relative to the reference. Initial participation is thus similar irrespective of social network topology (rows 2 and 3), but significantly lower in control (row 4). Furthermore, participation trends downward in treatment at a similar rate whatever the topology (rows 5 and 6), whereas, in control, declining participation in lattice requires a small negative adjustment (row 7). The other two networks, by contrast, call for significant positive adjustments (rows 8 and 9), sufficient to even reverse the decline in random regular network (Fig. 2). Significance: * P<0.05 and *** P<0.001. —, not applicable.

**Fig. 3.**
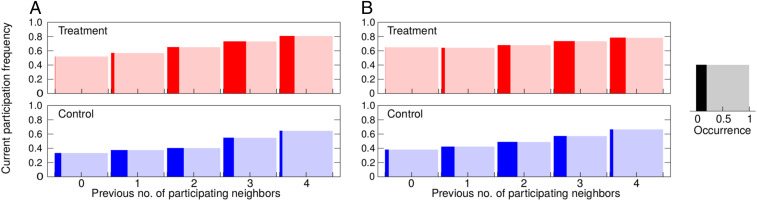
Increased participation is due to a cooperative environment created by free choice. Bar height shows participation in the current round depending on the number of participating neighbors in the previous round, whereas the width of color filling shows how frequent a particular number of previously participating neighbors was. For any value of this number, participation frequency is higher in treatment than in control (see also *SI Appendix*, Table S3). Situations with more cooperators also occur more often in treatment relative to control. The results for (*A*) lattice and (*B*) random regular network are qualitatively identical. Freedom of choice thus creates a more cooperative environment that improves participation in public goods provision. The results of a complementary analysis for degree 3 vs. degree 5 network, after separating the nodes of degree 3 from those of degree 5, is shown in *SI Appendix*, Fig. S5.

There is twofold evidence that the freedom of choice inspires a more cooperative environment. First, players more often find themselves surrounded by a larger number of participating neighbors (Fig. 3). With more participating neighbors in the preceding round, players are naturally inclined to participate more in the current round, and this is qualitatively the same in treatment and control. What is different, however, is that, even if the number of participating neighbors in the preceding round is the same in both treatment and control, the participation frequency is still higher in the former than the latter (Fig. 3). For example, with four participating neighbors in the preceding round, the current participation frequency in treatment is around 80%, whereas, under the same conditions in control, the participation frequency is only around 60%. Similar relations hold on the other end of the spectrum of possible situations, as indicated by the current participation frequency of 50 to 60% in treatment compared to 30 to 40% in control when no one participated in public goods provision in the preceding round. What we presume under the more cooperative environment is therefore 1) more neighborhoods with a larger number of participating players in treatment than control, and 2) a higher likelihood to participate in treatment than control even if the number of participating neighbors in the preceding round is the same.

**Fig. 4.**
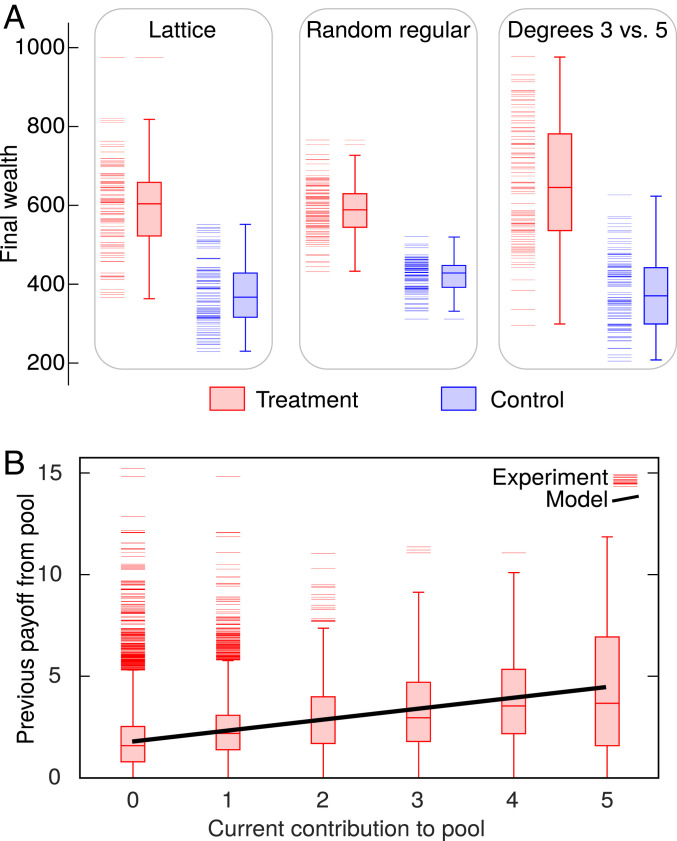
Freedom of choice adds value to public goods and motivates better provisioning. (*A*) Wealth generated in treatment is significantly higher than in control (*SI Appendix*, Tables S4 and S5). Moreover, in degree 3 vs. degree 5 network, players of degree 5 generate significantly more wealth (median values 536 and 788; Wilcoxon rank–sum test, test statistic W=33 with $n_{1}=n_{2}=50$, *P* value $<10^{-15}$). Raw data are represented by horizontal bars on the left, whereas the corresponding box-and-whisker plots on the right reveal the median (middle bar), the interquartile range (box height), the range that would encompass 99.3% of normally distributed data with the same mean and variance (whisker span), and outliers, if any. (*B*) In treatment, players seek better provision by investing more in pools that returned more previously. Roughly, every two additional points earned in the previous round increase the current investment by one point. The regression slope and its 95% CI are a=0.53 and [0.51,0.56], respectively. The intercept and its 95% CI are b=1.80 and [1.77,1.82], respectively. The coefficient of determination is $R^{2}=0.08$ (*F* test, test statistic F=2,055.6 with 24,009 degrees of freedom, *P* value $<10^{-15}$). We performed the analysis on raw data; binning is solely for visualization purposes. Shown are the results for lattice only, but a quantitatively similar trend holds for the other network types too: a=0.44 with [0.42,0.49] in random regular network, and a=0.44 with [0.41,0.46] in degree 3 vs. degree 5 network.

A more cooperative environment in treatment relative to control adds value to public goods (Fig. 4*A*). This result holds irrespective of topology, yet social networks do play a subtle role in shaping the distribution of final wealth. Lattice, for example, is less compact than random regular network of degree 4 in the sense that the former network has a larger diameter than the latter one. We find that this compactness is reflected in the distributions of final wealth, which are less dispersed (e.g., as measured by the interquartile range) in the case of random regular network than in the case of lattice. A varying player degree, by contrast, leads to the more dispersed distributions of final wealth because players with more connections benefit from additional freedom compared to their less connected counterparts. Moreover, the added value in treatment relative to control is, in part, due to better provisioning to public goods (Fig. 4*B*). When given the ability to direct their contributions, players seek to contribute to public goods with higher returns. The current contribution to a public good thus significantly depends on the preceding payoff from this public good (Fig. 4*B*), showing that the freedom of choice indeed motivates players to seek better provision, even if previous returns need not be the most reliable indicator of future performance.

**Fig. 5.**
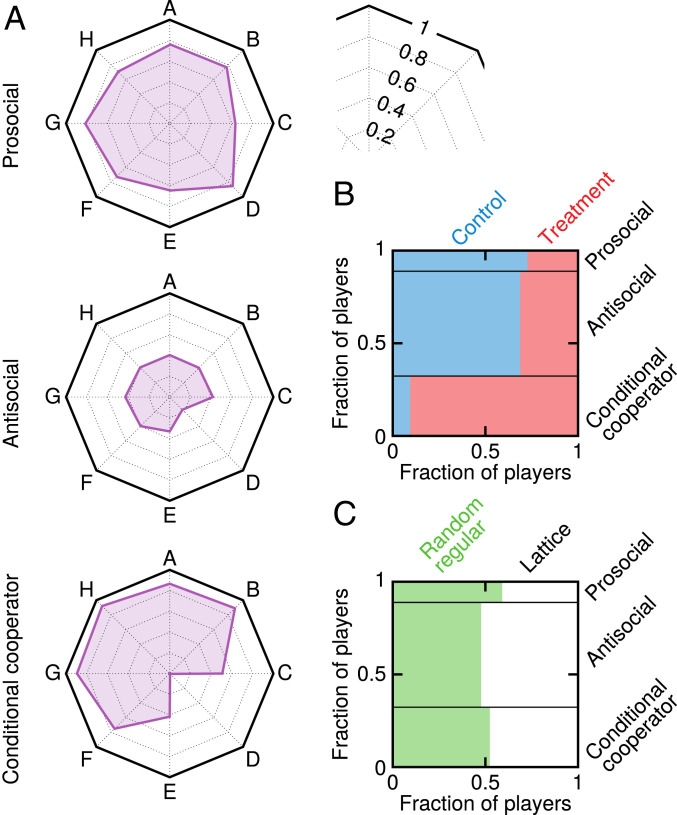
Decision-making is clearer when it mirrors preferences. (*A*) Shown are the behavioral phenotypes obtained using exploratory data mining based on eight empirical participation probabilities: A, overall; B, after participating in the preceding round; C, after nonparticipating in the preceding round; and D through H, after zero, one, two, three, or four neighbors participated in the preceding round. All probabilities except A are thus conditional on circumstances from the preceding round. Prosocial (antisocial) players regularly participate (refuse participation) in public goods provision irrespective of the circumstances. Conditional cooperators refuse participation when there are no other participants (i.e., cooperators) in the neighborhood. (*B* and *C*) Prosocial players are in the minority, comprising less than 15% of the population, while antisocial players dominate, comprising roughly 55% of the population. Between 65% and 70% of prosocial and antisocial players are found in control in which contributions are fixed. Interestingly, close to 90% of conditional cooperators come from treatment in which contributions are free (see also *SI Appendix*, Table S6). This indicates more clarity in the decision-making process when available options mirror preferences. Including degree 3 vs. degree 5 network into an analogous analysis yields similar conclusions, further showing that conditional cooperators are overabundant among players of degree 5, which underpins the good performance of these more connected players in terms of final wealth (*SI Appendix*, Fig. S6).

For a deeper understanding of the results, we turned to exploratory data mining to identify behavioral phenotypes. According to previous studies (24, 25), behaviors in game experiments are often classifiable into a small number of clusters, called behavioral phenotypes, that exhibit remarkable stability. Our data lead to similar conclusions. Namely, we identified three behavioral phenotypes (Fig. 5*A*) based on how players differ with respect to eight empirical participation probabilities: the overall participation probability, the participation probability after participating in the preceding round, the participation probability after nonparticipating in the preceding round, and the participation probability after zero, one, two, three, or four neighbors participated in the preceding round. Prosocial and antisocial phenotypes are the opposites with respect to their willingness to participate in public goods provision. The former regularly participate whereas the latter regularly refuse to participate regardless of the circumstances. The third type of players is different and exhibits behavior adaptive to the circumstances. Specifically, the players of this type refuse to participate when their neighbors do not participate and vice versa. The described behavior is thus reminiscent of conditional cooperators, that is, individuals whose cooperation depends on the cooperativeness of others (26). Prosocial players are in the minority, comprising less than 15% of the total, while antisocial players dominate, comprising roughly 55% of the total. Interestingly, around 65 to 70% of prosocial and antisocial players are found in control, whereas almost 90% of conditional cooperators are found in treatment (Fig. 5*B*). This indicates that the freedom of choice clarifies decision-making. Conversely, network configuration has no effect on the distribution of behavioral phenotypes, as evidenced by the nearly equal presence of all three phenotypes in both lattice and random regular network (Fig. 5*C*).

## Discussion

Herein, we have shown that letting players distribute their endowments freely increases participation in public goods provision and motivates better provisioning. This also entices larger player contributions and thus adds value to public goods. The added value is best seen in the larger final wealth in our free-contribution scenario. Intriguingly, increased participation in public goods provision happens from the very first round of the game rather than through gradual learning or optimization during the game, thus suggesting a sort of intuition on the part of the players about the tension encapsulated in the underlying social dilemma; see also ref. 27. Borrowing terminology from physics, choosing freely creates extra degrees of freedom in the system that serve to ease the underlying dilemma’s tension in favor of a more cooperative outcome.

Our results have multiple interesting implications. From a broader socioeconomic perspective, the results suggest that public goods might be better off when managed from the bottom up. Policy makers, for instance, could facilitate raising residential taxes by offering a portfolio of public goods for taxpayers to choose from. Such taxation schemes, by reflecting personal convictions, should be better at overcoming political election cycles or even austerity measures to finance long-term infrastructural projects. Furthermore, private companies might be able to motivate customers to pay premium prices if the premium could be directed toward a public good of the customers’ choosing. This would not only represent a step up in corporate social responsibility but could also help many seemingly profitable industries internalize environmental costs and thus remain profitable while doing justice to the environment. Car makers, for instance, might offset emissions by asking customers to direct premiums to preferred environmentally friendly projects.

Returning to a narrower perspective of social dilemma experiments, for the results to be truly useful in policy making, we need to understand the motivational basis for cooperative behavior. Especially in PGG experiments, prosocial tendencies as the driving force behind cooperation have been questioned (6). An alternative explanation has emerged instead, stating that player confusion in the form of misunderstanding of game instructions largely accounts for unexpected cooperativeness in PGGs (7), as well as for the decline of cooperation as the game progresses and players learn that their efforts are being undermined by defectors (28). Our experiment, by incorporating social networks rather than observing PGGs in isolation, enabled us to see that player participation in public goods provision is conditional on participation in the preceding time step by others (29). Players are simply more willing to participate if a larger number of neighbors participated in the previous round (Fig. 3). Players also tend to contribute more to public goods that yielded higher returns in the past (Fig. 4*B*). All of this suggests that player actions stem from calculated considerations, not confusion. Moreover, the proliferation of conditional cooperators in the free-contribution scenario (Fig. 5*B*) provides further evidence that players respond rationally to game conditions, because, in this scenario, signals from the surroundings are much clearer and players act accordingly, that is, when the surroundings are more cooperative, players cooperate more and vice versa. If there is any effect of confusion in the experiment, that happens when the surroundings emit conflicting signals, as is often the case in the fixed-contribution scenario. The data show that players then fall back to the “safe” antisocial option that precludes exploitation by others.

In sum, we find that players rely on a combination of an intuitive feel for the underlying social dilemma at the beginning of the game and signals from the surroundings to guide decisions in later rounds. Intuition thus merges with rationality to create a cooperative environment, whereas the safety of defection is mostly chosen as a way of coping with uncertainties. This view takes the focus away from the prevailing discussion on an inherent prosocial drive or confusion about the game rules. Instead, much in line with recent views on cognitive biases (30–32), it would seem that aspects of human cooperativeness remain hidden in simplified experimental environments and surface only in complex real-world ones, whose forces ultimately have shaped our decision-making faculties.

## Materials and Methods

### Aim and Goals.

A traditional focal point of social dilemma experiments has been human cooperativeness in isolated economic or evolutionary games. Recent theoretical studies, by contrast, turned to the role of complex social networks such that players within their well-defined neighborhoods take part in multiple games simultaneously. We aimed to merge the two perspectives with a twofold goal, that is, to determine 1) whether the topology of social networks affects participation in public goods provision and 2) how adding a natural degree of freedom that lets players direct their contributions to preferred public goods affects decision-making. To do so, we devised an experimental protocol that envisioned recruiting a pool of players whom we randomly assigned to one of six experimental setups. With respect to social networks, the options included a lattice, a random regular network of degree 4, and a random network in which one half of the nodes have degree 3 and the other half have degree 5. With respect to the freedom of choice, players in experimental control could only decide whether or not to participate in public goods provision, whereas players in experimental treatment could preferentially direct their contributions to more-desirable public goods. We thus used control groups to determine the baseline participation frequency in the first and following rounds, comparable with other public goods experiments. Treatment groups served to determine participation rates when players could freely choose how and where to distribute their contributions.

### Experimental Setup.

We embedded the classical PGG into complex social networks. Accordingly, each player engaged in more than one PGG and could contribute to more than one pool, depending on their network neighborhood. Players in lattice and random regular network with degree 4 could contribute to a total of five pools in every round of the game. This is because players engaged in one PGG centered around themselves, and four PGGs centered around each of the first neighbors. Similarly, in degree 3 vs. degree 5 network, half of the players could contribute to four and the other half to six pools. When players in control groups decided to contribute, one endowment unit would be contributed on their behalf to each of the pools, except in degree 3 vs. degree 5 network in which 1.25 (0.83) units would be contributed on behalf of players of degree 3 (degree 5), amounting to a total of five units per player. In treatment groups, by contrast, players who decided to contribute could freely direct their contributions to preferred common pools, with a minimal contribution set to 0.01 units and the total amount again equal to five units. The compounding factor equaled four in all games.

### Player Recruitment and Implementation.

We held 12 sessions of the experiment at the behavioral economics laboratory of the Yunnan University of Finance and Economics in Kunming city, China, between May and December 2018. For each session, we randomly selected voluntary participants from a pool of students (*SI Appendix*, Table S1). Volunteers showing up on the day of the experiment were directed to isolated computer cubicles where they read on-screen instructions (*SI Appendix*, *Supplementary Methods*). Thereafter, a pregame test checked whether the instructions had been properly understood; those who failed to answer the test were asked to reread the instructions and then retake the test. Gameplay started with two exercise rounds, and continued for 50 rounds that counted toward the final score. We kept the total number of rounds undisclosed. The initial endowment was 50 points. In each round, players had 30 s to make a decision using a custom gameplay interface (*SI Appendix*, Fig. S2); a failure to do so would trigger the default choice of no-participation. This was followed by a period of another 30 s to inspect the results (*SI Appendix*, Fig. S3). We developed the gameplay interface in the o-Tree platform for laboratory, online, and field experiments (33). At the end of the game, the final result was turned into a monetary payout at a rate of ¥0.2 for one point. Players also received a show-up fee of ¥15. The average payout was ¥115.87, ranging from ¥15 to ¥216.45.

### Ethics Statement.

The experiment was approved by the Ethics Committee on the Use of Human Participants in Research of the Yunnan University of Finance and Economics. We obtained informed consent from all volunteers.

### Data Availability and Analyses.

The datasets generated and analyzed herein are available in the Open Science Framework repository, doi:10.17605/OSF.IO/H4ZK5 (34). Among standard statistical techniques, we used 1) Fisher’s test for contingency tables and 2) the two-way ANOVA for the dependence of one continuous dependent variable on two categorical independent variables. Importantly, all hypothesis tests performed on the available data are reported without exception, irrespective of the significance of results. To characterize the time evolution of participation in public goods provision, we additionally employed time series analysis to identify trends, stationarity, and autocorrelations. Finally, to extract behavioral phenotypes from the data (24, 25), we employed a form of unsupervised machine learning called cluster analysis.

## Supplementary Material

Supplementary File
