# Supplementary material for: Freedom of choice adds value to public goods
Source: Proc Natl Acad Sci U S A. 2020 Jul 13;117(30):17516–21. doi: 10.1073/pnas.1921806117 (PMC7395457; doi:10.1073/pnas.1921806117)
Supplement: Supplementary File [file pnas.1921806117.sapp.pdf]

Supplementary Information for

Freedom of choice adds value to public goods

Lei Shi, Ivan Romić, Yongjuan Ma, Zhen Wang, Boris Podobnik, H. Eugene Stanley, Petter Holme, Marko Jusup

Corresponding authors: Zhen Wang, H. Eugene Stanley, and Marko Jusup
E-mail: w-zhen@nwpu.edu.cn (Z.W.), hes@bu.edu (H.E.S.), and mjusup@gmail.com (M.J.)

This PDF file includes:

Supplementary text
Figs. S1 to S6
Tables S1 to S7

www.pnas.org/cgi/doi/10.1073/pnas.1921806117

Supporting Information Text

Supplementary methods

Here follows an English translation of gameplay instructions as displayed to volunteers in experimental treatment before the beginning of the game. We minimally adjusted the text where necessary to suit the needs of experimental control.

Instructions

Welcome to our social-dilemma game experiment!

Please read the following instructions carefully. If you experience any problems during the game, please raise your hands and our expert staff will help you. This is an anonymous experiment; a computer system will assign everyone a random ID number that cannot be linked back to you. During the game, please refrain from attempts to communicate with other players.

1. Background and objective: Decision making in life often involves a dilemma between personal interests and common good. The objective of this game experiment is to test decision-making patterns in the presence of such dilemmas. You and four other players will thus be asked at the same time whether and how much you wish to contribute to a common pool. After you post your decisions, the accumulated contribution will be multiplied by a factor of four and divided equally among all five players (Fig. 1).

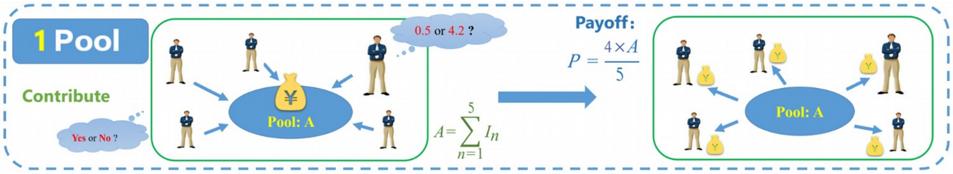

Figure 1. Schematic diagram of gameplay with a single pool. Five players are potential contributors who decide at the same time whether and how much (I_n, n=1,...,5) to contribute to the pool. The accumulated contribution (A) is multiplied by a factor of four and equally divided among all five potential contributors such that everyone receives the same payoff (P).

2. Gameplay rules: During the game, you will be located somewhere on a two-dimensional lattice, and act as a potential contributor to five different pools at the same time; one pool will be centered around you while the remaining four pools will be centered around each of your four neighbors (Fig. 2). Everyone will be given 50 starting points as an endowment. The game consists of an undetermined number of rounds. In each round, you need to decide whether you wish to contribute to pools within your reach, and then decide how much you wish to contribute. There is no cost if you decide not to contribute. If you do decide to contribute, the total contribution must equal five points, meaning that a minimum contribution to any of the pools is 0.01 and the maximum contribution is 4.96. Pools are independent of one another. Accumulated contributions in each of the pools will be multiplied by a factor of four, and distributed equally among five potential contributors. Your total payoff in each round will be the sum of payoffs from each of the five pools (Fig. 3).

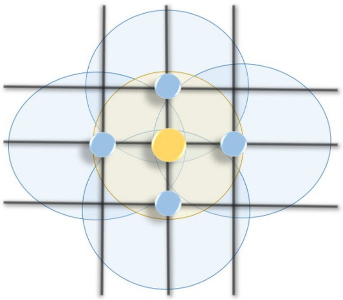

Figure 2. Placement of players on a two-dimensional lattice. Imagining yourself in the center, there will be four other players who are your nearest neighbors. All of you together are potential contributors to one pool (light yellow) centered around yourself. However, there will also be four other pools (light blue) centered around each of your neighbors to which you will be able to contribute as well.

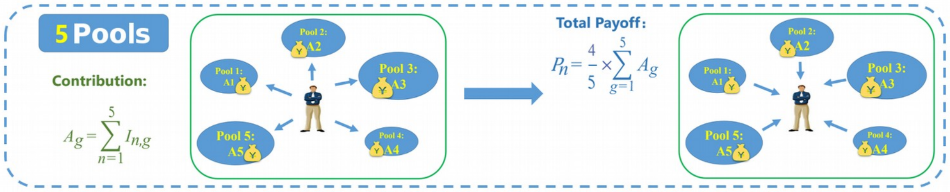

Figure 3. Schematic diagram of gameplay with five pools. Every player is a potential contributor to five pools. The accumulated contributions (A_g, g=1,...,5) are multiplied by a factor of four and equally divided among five potential contributors. Any player's total payoff (P_n) will be the sum of payoffs from pools they are contributing to.

3. Computer interface: Gameplay happens via a custom computer interface consisting of two screens:
• On the investment screen, you will choose whether to contribute or not, and then select the amount to contribute to each of the five pools within your reach. A time period of 30 seconds is alloted to this task. When you finish posting your decisions, clicking the "Next" button will take you to the next screen. If you fail to click on the button within the alloted time, the system will choose the default option of no-contribution and automatically proceed to the next screen.
• On the result screen, you will be able to inspect key information for a period of 30 seconds. This includes the total amount contributed to each of the five pools, individual contributions to the pools made by you and your neighboring players, payoffs that you and your neighbors obtained from the pools, and the amounts contributed by your neighbors to the pools that you share with them. You will also be able to see your wealth accumulated throughout the game. When you finish inspecting the results, clicking the "Next" button will take you to the next screen. If you fail to click on the button within the alloted time, the system will automatically proceed to the next screen.

4. Monetary payout: When the game finishes, you will be able to see your final wealth. Our staff will convert this wealth into a real monetary payout at a rate of ¥0.2 per 1 point. You will also obtain a show-up fee of ¥15 irrespective of your performance during the experiment.

After reading the instructions, we asked volunteers to take part in a brief pregame quiz. Those volunteers who solved the quiz correctly were cleared for gameplay, whereas those who made mistakes were directed to reread the instructions and then retake the quiz. Gameplay started only after everyone answered the quiz successfully.

Pregame quiz

ONE POOL: Five players, including you, have a chance to contribute to a public pool. The contributions by others are 4.6, 1.0, 0.0, 0.0, while your contribution is 2.0. According to the game rules, the total payoff to be shared between the players is ______ points, and each player will individually get ______ points.

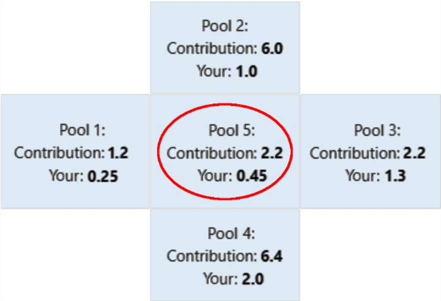

Figure 4. There are five public pools in your neighborhood, one pool is centered around you, while the other four are each centered around one of your neighbors. Shown are the total contributions to all five pools and your part immediately below.

FIVE POOLS: Consider the situation in Figure 4 assuming that you started the current round of the game with a wealth of 50 points. In this round, you will obtain payoff in the amount of ______ points, and your total wealth will increase to ______ points.

Supplementary figures

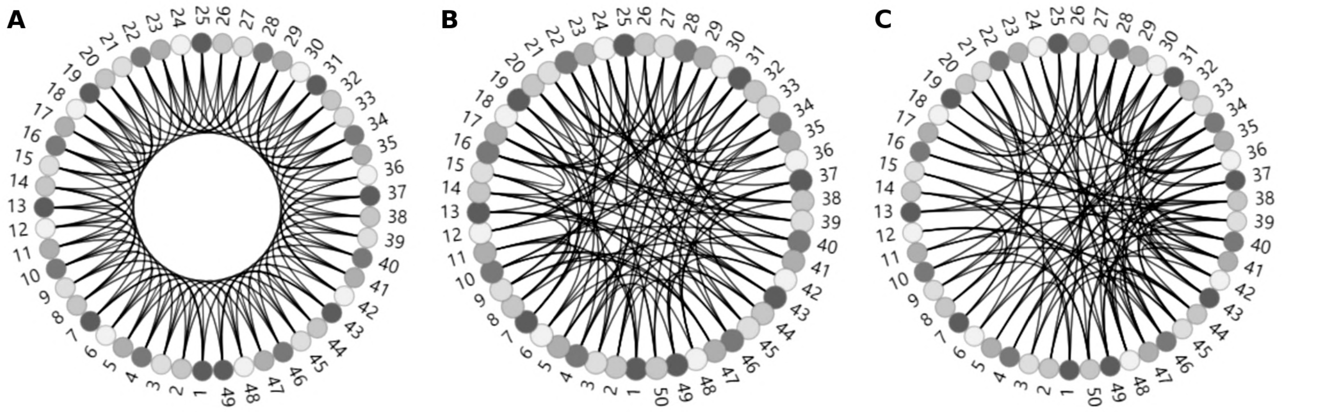

Fig. S1. Three network configurations used in the experiment. A, Lattice is recognizable by its regular structure and a large diameter—defined as the length of the longest among all shortest paths in the network. This is evident from a distinct lack of links between near-diametrically opposing nodes. **B,** Random regular network of degree four, unlike lattice, has many links between near-diametrically opposing nodes. This tends to reduce the diameter by shortening all paths in the network, including the longest among all shortest paths. The network was constructed from lattice by randomly selecting two links, say, between nodes a–b and c–d, and then randomly rewiring to create either links a–c and b–d, or links a–d and b–c. Rewired links could not be rewired again. **C,** Random degree 3 vs. 5 network may appear similar to random regular network, but upon a closer inspection, it is possible to distinguish that nodes 1 to 25 with degree 3 are less densely interlinked than nodes 26 to 50 with degree 5. The network was constructed from a realization of regular random network by randomly selecting one link from each of the nodes between 1 and 25, and then rewiring it to a randomly selected node between 26 and 50. Each of the latter nodes could accept only one link.

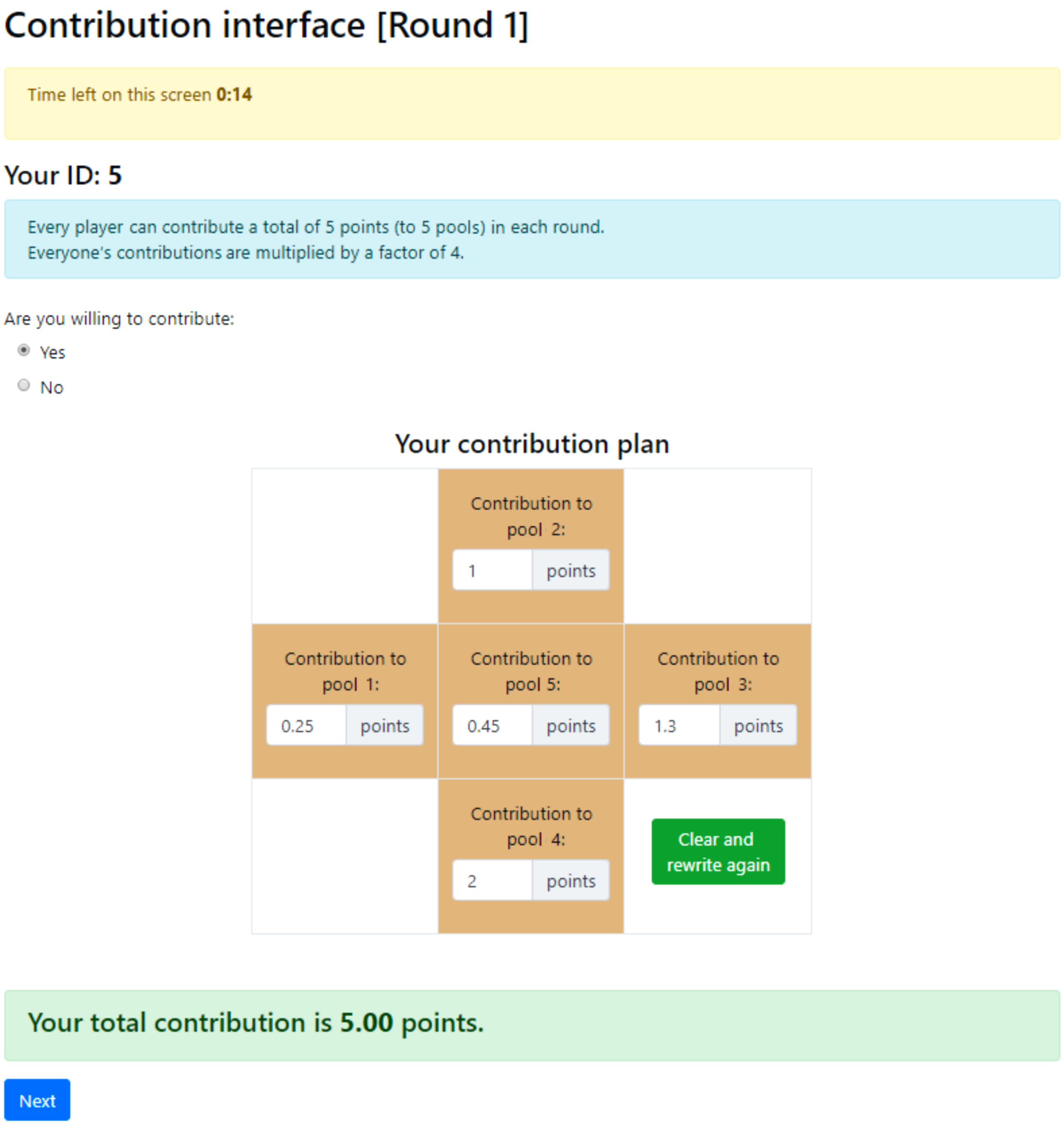

Fig. S2. Gameplay computer screen used in experimental treatment. Players used this screen to decide whether to participate in public goods provision or not, as well as to distribute their endowments to preferred public goods. In control treatment, once a decision to participate had been made, one endowment unit was distributed to each of the public goods within a player's reach.

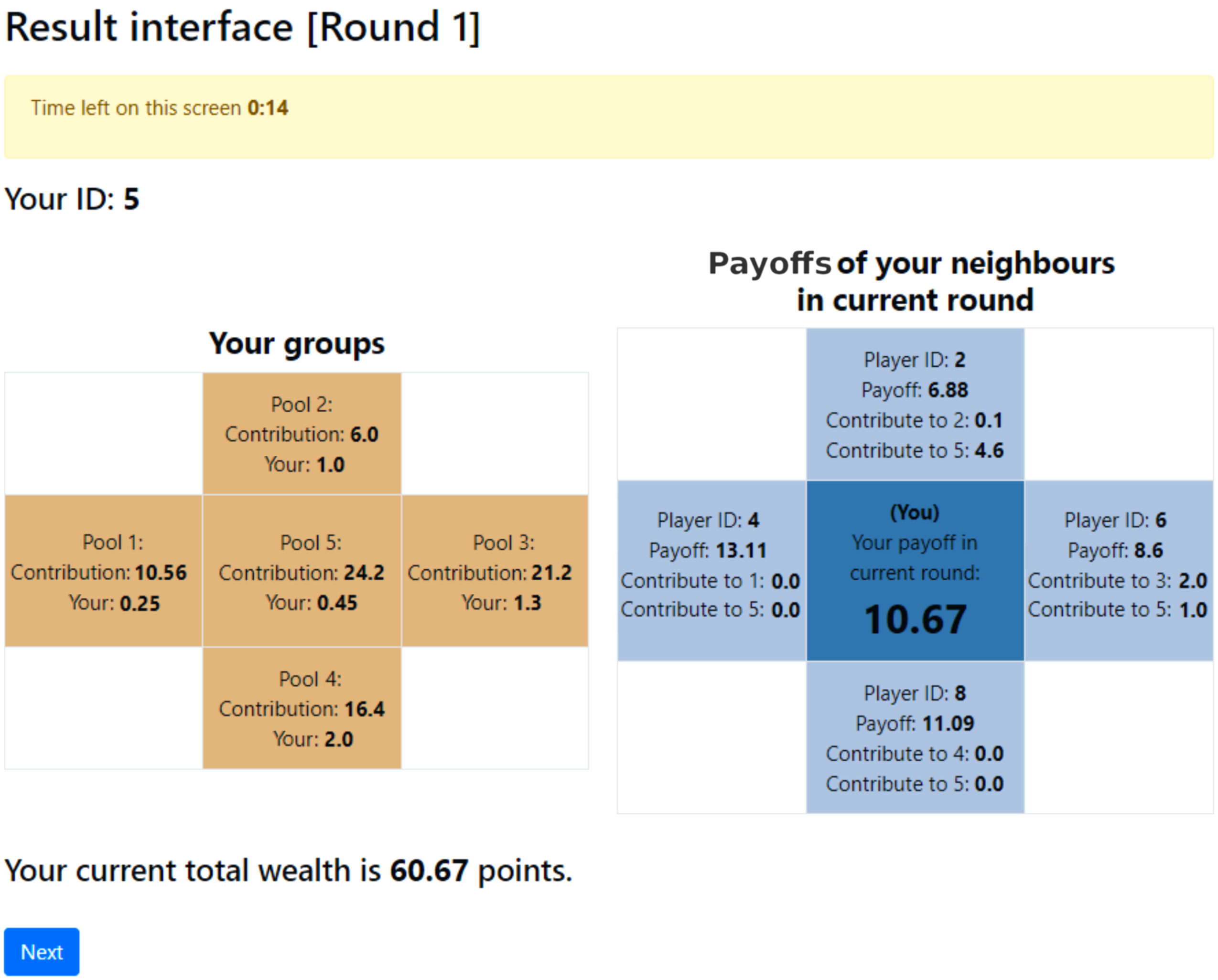

Fig. S3. Result computer screen displayed in experimental treatment. Before proceeding to the next round, players could examine detailed information on their own performance and the performance of their neighbors. Specifically, for each of the PGGs that the player participates in, the displayed information included pool sizes in the current round together with the amounts contributed by the player to each of these pools (left), and own and neighbor payoffs in the current round together with the relevant neighbor contributions (right). An analogous screen was used in control, but there contributions could only amount to zero or one.

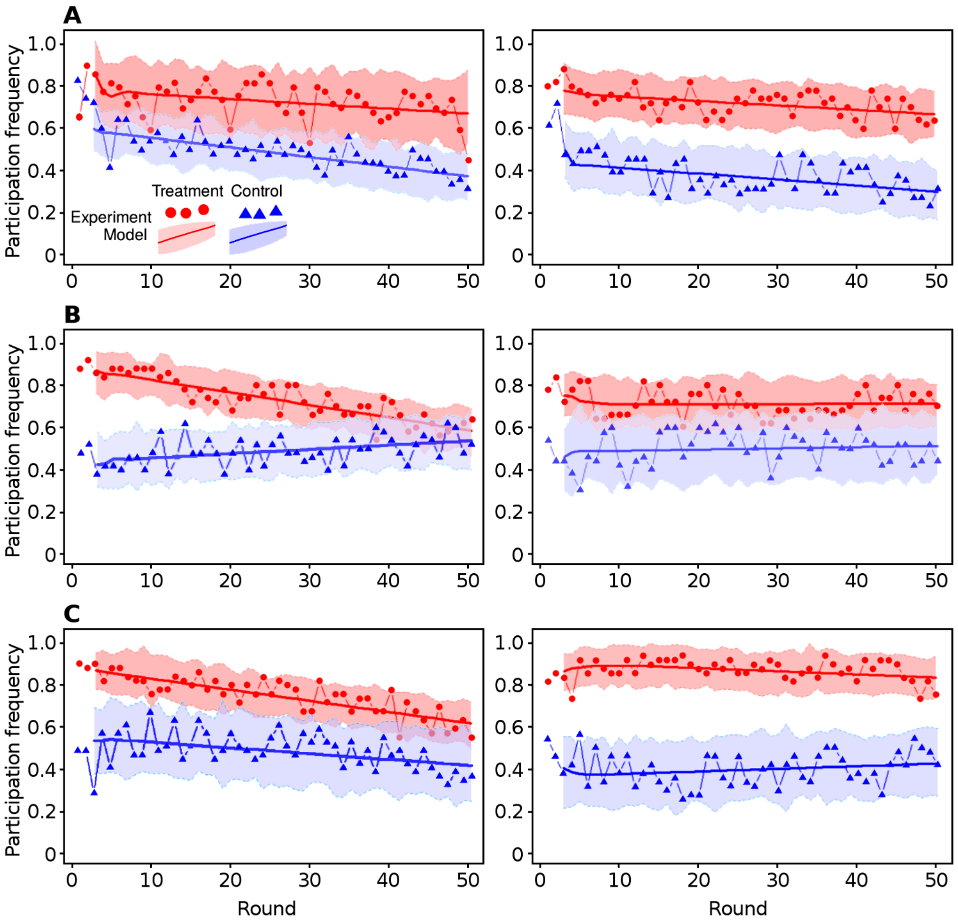

Fig. S4. Participation in public goods provision may worsen, stabilize, or improve over time in individual sessions of the experiment. Shown is the time evolution of the average participation in game experiments, but now for each replicate separately. Raw datasets (blue triangles for control and red circles for treatment) are accompanied by a fitted time-series model that reveals (i) trends, (ii) stationarity, and (iii) auto-correlations. The model's 95 % confidence bands are also shown. **A,** In lattice, all four replicates exhibit slow-but-persistent, statistically significant, negative trend in participation. **B,** In random regular network, we recorded one instance of a negative trend (left panel, treatment), one instance of a positive trend (left panel, control), and two instances of no trend in participation (right panel). **C,** In degree 3 vs. 5 network, we had two instances of a negative trend (left panel) and two instances of no trend in participation (right panel). All time series are stationary. These results reveal a considerable variation in participation trends between individual sessions of the experiment, but the freedom of choice ensures without fail higher participation frequencies for prolonged periods of time.

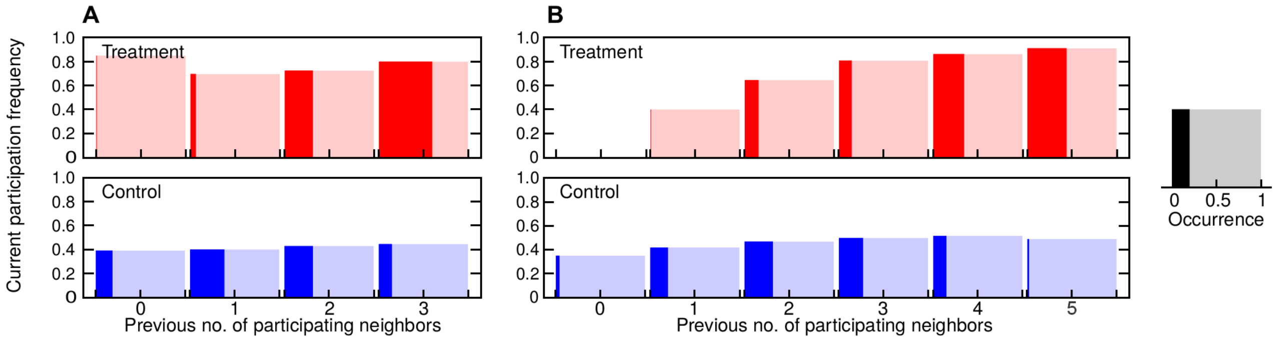

Fig. S5. Participation patterns in degree 3 vs. 5 network echo those in other networks. Bar height shows participation in the current round depending on the number of participating neighbors in the previous round, whereas the width of color filling shows how frequent a particular number of previously participating neighbors was. For nearly all values of this number, participation frequency is higher in treatment than in control. Situations with more cooperators also occur more often in treatment relative to control. **A, B,** Nodes of both degree three (left) and degree five (right) display similar cooperation patterns. Some situations occur rarely, e.g., zero participating neighbors in treatment or five participating neighbors in control, making the corresponding estimates of participation frequency less reliable.

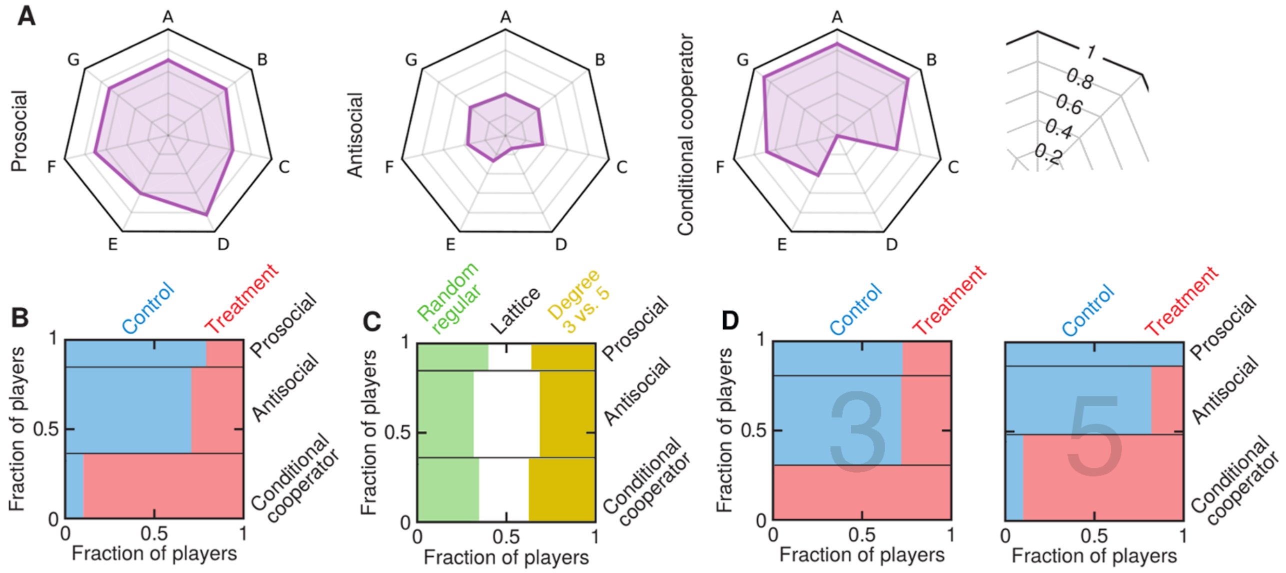

Fig. S6. Behavioral phenotypes in all three networks. Shown are the results of exploratory data mining analogous to that in Fig. 5 of the main text, but now with degree 3 vs. 5 network included and with seven instead of eight empirical participation probabilities taken into account: A—overall, B—after participating in the preceding round, C—after non-participating in the preceding round, and D through G—after zero, one, two, or three or more neighbors participated in the preceding round. All probabilities except A are thus conditional on circumstances from the preceding round. **A, B,** Adding degree 3 vs. 5 network into the mix leaves behavioral phenotypes and their prevalence in the system largely unaltered. Prosocial players are in minority, while antisocial players are in majority, and both of these phenotypes appear mostly in control. Conditional cooperators appear mostly in treatment. **C,** Social network has little influence on the prevalence of behavioral phenotypes. Prosocial players and conditional cooperators are slightly underrepresented, whereas antisocial players are slightly overrepresented in lattice. **D,** Players of degree three and five qualitatively follow the described patterns, although there is an important difference between them. Specifically, an increased prevalence of conditional cooperators among players of degree five comes at the expense of antisocial phenotype. This helps players of degree five to accumulate more wealth than players of degree three during a typical treatment session of the experiment.

Supplementary Tables

Table S1. Basic demographic information on volunteers.

Experimental setup		Rounds	Players	Women (%)	Date	Mean age (SD)
Lattice	*Treatment*	50	98	56	2018.05.11.	20.5 (0.99)
					2018.05.18.	20.0 (1.00)
	Control	50	98	60	2018.05.12.	21.0 (1.00)
					2018.05.19.	20.0 (1.00)
Random regular	*Treatment*	50	100	59	2018.10.23.	18.6 (0.94)
					2018.12.07.	18.6 (0.72)
	Control	50	100	65	2018.10.24.	18.7 (0.81)
					2018.12.07.	18.3 (0.75)
Degree 3 vs. 5	*Treatment*	50	100	72	2018.10.27.	18.7 (1.03)
					2018.12.08.	18.5 (0.75)
	Control	50	100	64	2018.10.28.	18.4 (0.67)
					2018.12.13.	18.6 (0.63)

Experimental setup was defined by specifying a network configuration (lattice, random regular network of degree four, or random network with the average degree of four) and a contribution type (fixed in control or free in treatment). The total number of setups was thus six. Each experimental setup was replicated twice for a total of 12 sessions of the experiment. Within practical limitations, we aimed at recruiting a gender-balanced sample of volunteers across setups. We separately examined the influence of gender as a confounding factor for completeness (Supplementary Table S7).

Table S2. Time-series analysis of participation in public goods provision recorded in the experiment.

Summary (model)	Coefficients		Estimate	t-statistic	p-value
**Lattice**					
Treatment	*Constant*	α_1	0.606	4.035	**<10^{-3}**
	Trend	α_2	–0.002	–2.848	**0.007**
$R^2_{\mathrm{adj}} = 0.272$	*Stationarity*	α_3	–0.780	–4.064	**<10^{-3}**
	Auto-correlative	α_4	0.156	1.069	0.291
Control	*Constant*	α_1	0.398	5.756	**<10^{-6}**
	Trend	α_2	–0.003	–4.218	**<10^{-3}**
$R^2_{\mathrm{adj}} = 0.462$	*Stationarity*	α_3	–0.774	–6.197	**<10^{-6}**
	Auto-correlative	α_4	0.098	0.831	0.411
**Random regular**					
Summary (model)	Coefficients		Estimate	t-statistic	p-value
Treatment	*Constant*	α_1	0.754	4.519	**<10^{-4}**
	Trend	α_2	–0.003	–3.594	**<10^{-3}**
$R^2_{\mathrm{adj}} = 0.486$	*Stationarity*	α_3	–0.941	–4.619	**<10^{-4}**
	Auto-correlative	α_4	–0.048	–0.331	0.742
Control	*Constant*	α_1	0.315	3.890	**<10^{-3}**
	Trend	α_2	0.001	1.801	0.079
$R^2_{\mathrm{adj}} = 0.280$	*Stationarity*	α_3	–0.686	–3.937	**<10^{-3}**
	Auto-correlative	α_4	0.043	0.287	0.776
**Degree 3 vs. 5**					
Summary (model)	Coefficients		Estimate	t-statistic	p-value
Treatment	*Constant*	α_1	0.525	2.831	**0.007**
	Trend	α_2	–0.002	–2.878	**0.006**
$R^2_{\mathrm{adj}} = 0.443$	*Stationarity*	α_3	–0.589	–2.822	**0.007**
	Auto-correlative	α_4	–0.334	–2.189	0.034
Control	*Constant*	α_1	0.638	6.420	**<10^{-7}**
	Trend	α_2	–0.001	–2.175	0.035
$R^2_{\mathrm{adj}} = 0.550$	*Stationarity*	α_3	–1.389	–6.510	**<10^{-7}**
	Auto-correlative	α_4	0.272	1.895	0.065

We fitted equation $C_t - C_{t-1} = \alpha_1 + \alpha_2 t + \alpha_3 C_{t-1} + \alpha_4(C_{t-1} - C_{t-2}) + \epsilon_t$ to the data displayed in Fig. 2 of the main text, where α_i, $i = 1, 2, 3, 4$ are the regression coefficients, and ϵ_t is a normally distributed error term with a zero mean and an unknown variance. The regression coefficients have the following interpretation: α_1 is a constant term, $\alpha_2 \neq 0$ indicates trend, $\alpha_3 < 0$ indicates stationarity, and $\alpha_4 \neq 0$ indicates auto-correlation. The results show that a small negative trend in participation is predominant, but not a rule (see also Supplementary Fig. S4). All time series are stationary without any or, at best, with very weak positive auto-correlation.

Table S3. Contingency tables to compare participation in public goods provision between treatment and control.

**Lattice**						
Participating neighbors		0	1	2	3	4
Treat.	*C*	19 (12) [3.5]	170 (121) [19.6]	699 (543) [44.5]	1498 (1379) [10.3]	1091 (1057) [1.1]
	D	17 (24) [1.8]	128 (177) [13.4]	373 (529) [45.8]	548 (667) [21.2]	259 (293) [4.0]
Cont.	*C*	198 (205) [0.2]	563 (612) [3.9]	607 (763) [31.7]	522 (641) [22.1]	163 (197) [5.9]
	D	394 (387) [0.1]	940 (891) [2.7]	897 (741) [32.6]	429 (310) [45.7]	89 (55) [21.4]
Chi-square statistic		5.61	39.5	155	99.2	32.5
Fisher test p-value		0.029	$<10^{-9}$	$<10^{-15}$	$<10^{-15}$	$<10^{-7}$
**Random regular**						
Participating neighbors		0	1	2	3	4
Treat.	*C*	11 (7) [2.8]	203 (148) [20.4]	797 (662) [27.7]	1412 (1293) [11]	1132 (1102) [0.8]
	D	6 (10) [1.8]	115 (170) [17.8]	384 (519) [35.2]	520 (639) [22.2]	320 (350) [2.5]
Cont.	*C*	131 (135) [0.1]	515 (570) [5.2]	894 (1029) [17.8]	677 (796) [17.9]	201 (231) [3.9]
	D	214 (210) [0.1]	709 (654) [4.6]	943 (808) [22.7]	513 (394) [36.1]	103 (73) [12.1]
Chi-square statistic		4.86	48.0	103	87.2	19.3
Fisher test p-value		0.040	$<10^{-11}$	$<10^{-15}$	$<10^{-15}$	$<10^{-4}$

Situations when players are surrounded by zero, one, two, three, or four participating neighbors in the preceding round are considered separately. Values indicate the number of times an event has occurred, values in round brackets are the expected occurrences, and values in square brackets are the contributions to the chi-squared statistic. Labels C and D stand for participation (i.e., cooperation) and non-participation (i.e., defection) in the current round, respectively. Behavior in treatment is generally much more cooperative than in control, irrespective of the network configuration. We did not analyze degree 3 vs. 5 network in this manner because of the two differing neighborhood sizes.

Table S4. Analysis of variance (ANOVA) for the dependence of final wealth on network configuration (lattice, random regular, or degree 3 vs. 5) and contribution type (fixed or free).

Term	d. f.[a]	SS[b]	MS[c]	F statistic	p-value	p-value[d]
Network configuration	2	136647	68324	7.059	$<\mathbf{10^{-3}}$	0.012
Contribution type	1	7541744	7541744	779.169	$<\mathbf{10^{-15}}$	$<\mathbf{10^{-15}}$
Configuration × Type	2	283814	141907	14.661	$<\mathbf{10^{-6}}$	$<\mathbf{10^{-6}}$
Residuals	590	5710737	9679	–	–	–

[a] Degrees of freedom
[b] Sum of squares
[c] Mean squares
[d] Robust ANOVA

ANOVA indicates, and robust ANOVA confirms, that final wealth strongly depends on contribution type, which is fixed in control and free in treatment. The dependence on network configuration is much weaker but still significant. For robust ANOVA *post-hoc* comparisons see the accompanying Supplementary Table S5.

Table S5. Robust *post-hoc* comparisons for the dependence of final wealth on network configuration (lattice, random regular, or degree 3 vs. 5) and contribution type (fixed or free).

Comparison			Difference	LCB[a]	UCB[b]	p-value
Lattice	vs.	*Random regular*	–75.311	–133.017	–12.682	**0.006**
Lattice	vs.	*Degree 3 vs. 5*	–54.579	–99.049	–13.856	**0.002**
Random regular	vs.	*Degree 3 vs. 5*	20.732	–34.981	66.824	0.186
Treatment	vs.	*Control*	–677.892	–721.500	–623.317	**$<10^{-3}$**
Lattice Treat. *Rand. reg. Cont.*	vs.	*Lattice Cont.* *Rand. reg. Treat.*	59.501	–2.922	116.591	0.021
Lattice Treat. *Deg. 3 vs. 5 Cont.*	vs.	*Deg. 3 vs. 5 Treat.* *Lattice Cont.*	–54.450	–93.663	–8.674	**0.003**
Rand. reg. Treat. *Deg. 3 vs. 5 Cont.*	vs.	*Deg. 3 vs. 5 Treat.* *Rand. reg. Cont.*	–113.951	–156.746	–57.084	**$<10^{-3}$**

[a]Lower confidence bound
[b]Upper confidence bound

Post-hoc comparisons clearly emphasize the dependence of final wealth on contribution type, which is fixed in control and free in treatment. The importance of network configuration is primarily due to an overall lower final wealth in lattice than in the two other configurations. The interaction between network configuration and contribution type is significant mostly due to an exceptionally large final wealth in degree 3 vs. 5 network when contributions are free. This exceptional wealth is earned by players of degree five who take part in more PGGs than the players of degree three and four.

Table S6. Contingency tables to compare the abundance of behavioral phenotypes between treatment and control, as well as between lattice and random regular network.

Experimental setup	Prosocial	Antisocial	Conditional cooperator	Chi-square statistic	Fisher test p-value
Treatment	12 (22.00) [4.55]	70 (112.00) [15.75]	116 (64.00) [42.25]	125.09	$<\mathbf{10^{-15}}$
Control	32 (22.00) [4.55]	154 (112.00) [15.75]	12 (64.00) [42.25]		
Lattice	18 (21.78) [0.66]	117 (110.87) [0.34]	61 (63.35) [0.09]	2.142	0.34
Rand. reg.	26 (22.22) [0.64]	107 (113.13) [0.33]	67 (64.65) [0.09]		

Values indicate the actual number of players exhibiting a particular behavioral phenotype, values in round brackets are the expected abundances, and values in square brackets are the contributions to the chi-squared statistic. When treatment is compared to control, all behavioral phenotypes substantially deviate from the expected abundances, thus contributing to a highly significant result. The freedom of choice suppresses the antisocial phenotype and promotes conditional cooperators. When lattice is compared to random regular network, all behavioral phenotypes are fairly close to the expected abundances. Network configuration therefore has no distinguishable impact on behavior.

Table S7. Role of gender as a confounding factor.

Log-linear model	Interactions	AIC[a]
Complete independence	none	1603.1
Joint independence I	*experimental setup* × *gender*	1552.5
Joint independence II	*experimental setup* × *action*	175.1
Joint independence III	*gender* × *action*	1590.8
Conditional independence I	*experimental setup*×*gender*<br>*experimental setup*×*action*	124.4
Conditional independence II	*experimental setup*×*gender*<br>*gender*×*action*	1540.1
Conditional independence III	*experimental setup* × *action*<br>*gender* × *action*	162.7
Homogeneous association	all two-way	**90.9**
Saturated	all	92.6

[a] Akaike information criterion

Here, we used log-linear models to explain cooperation and defection counts in a three-way table formed by categorical variables *experimental setup*, *gender*, and *action*. Compared to the most basic model of complete independence, adding the *experimental setup*×*action* interaction greatly improves performance in terms of the AIC. This is because players are generally much more cooperative in treatment relative to control. That two interactions involving gender also contain some explanatory power is evidenced by the fact that the model of homogeneous association has the lowest AIC value. The *gender*×*action* interaction is simply because women outnumbered men in the recruited pool of students. More importantly, the *experimental setup*×*gender* interaction is due to lower cooperativeness of women (44 %) in control relative to men (49 %). We could therefore expect that all-women sessions produce somewhat stronger effect than all-men sessions of the experiment.
